# Supplementary material for: Hot spot prediction in protein-protein interactions by an ensemble system
Source: BMC Syst Biol. 2018 Dec 31;12(Suppl 9):132. doi: 10.1186/s12918-018-0665-8 (PMC6311905; doi:10.1186/s12918-018-0665-8)
Supplement: Supplementary file 2 — All relevant MATLAB code. A simple Matlab implement of our predictor. (ZIP 19,742 kb) [file 12918_2018_665_MOESM2_ESM.zip › Additional file 2/Read ASA/ASA website/dbMPIKT/1DFJI.html]

 NetSurfP - Protein Surface Accessibility and Secondary Structure Predictions

  

|  |  |  |
| --- | --- | --- |
|  |  | NetSurfP - Protein Surface Accessibility and Secondary Structure Predictions  Technical University of Denmark |

  


---

```
# For publication of results, please cite:
# A generic method for assignment of reliability scores applied to solvent accessibility predictions.
# Bent Petersen, Thomas Nordahl Petersen, Pernille Andersen, Morten Nielsen and Claus Lundegaard
# BMC Structural Biology 2009, 9:51 doi:10.1186/1472-6807-9-51
#
# Column 1: Class assignment - B for buried or E for Exposed - Threshold: 25% exposure, but not based on RSA
# Column 2: Amino acid
# Column 3: Sequence name
# Column 4: Amino acid number
# Column 5: Relative Surface Accessibility - RSA
# Column 6: Absolute Surface Accessibility
# Column 7: Z-fit score for RSA prediction
# Column 8: Probability for Alpha-Helix
# Column 9: Probability for Beta-strand
# Column 10: Probability for Coil
  X  1DFJI                  1    0.000   0.000   0.000   0.000   0.000   0.000
B M  1DFJI                  2    0.163  32.556  -0.859   0.005   0.015   0.979
E N  1DFJI                  3    0.413  60.536   0.696   0.003   0.718   0.279
B L  1DFJI                  4    0.069  12.671   0.261   0.001   0.959   0.040
E D  1DFJI                  5    0.342  49.210   1.120   0.001   0.959   0.040
B I  1DFJI                  6    0.070  13.042   0.050   0.001   0.959   0.040
E H  1DFJI                  7    0.387  70.468  -0.387   0.003   0.718   0.279
E C  1DFJI                  8    0.591  82.976  -1.500   0.004   0.420   0.576
B E  1DFJI                  9    0.286  49.964   0.227   0.004   0.197   0.799
E Q  1DFJI                 10    0.531  94.837  -0.032   0.004   0.138   0.858
B L  1DFJI                 11    0.064  11.682  -0.454   0.004   0.138   0.858
E S  1DFJI                 12    0.358  41.911   0.288   0.018   0.047   0.935
E D  1DFJI                 13    0.500  72.050  -1.549   0.018   0.019   0.964
E A  1DFJI                 14    0.642  70.715   0.719   0.782   0.003   0.216
B R  1DFJI                 15    0.147  33.594   0.145   0.858   0.002   0.139
B W  1DFJI                 16    0.031   7.480   0.262   0.858   0.002   0.139
E T  1DFJI                 17    0.466  64.579   1.683   0.970   0.001   0.030
E E  1DFJI                 18    0.368  64.342   1.189   0.970   0.001   0.030
B L  1DFJI                 19    0.028   5.182  -0.012   0.970   0.001   0.030
B L  1DFJI                 20    0.089  16.241  -1.055   0.970   0.001   0.030
E P  1DFJI                 21    0.624  88.617   1.164   0.970   0.001   0.030
B L  1DFJI                 22    0.246  45.116  -0.593   0.923   0.002   0.076
B L  1DFJI                 23    0.060  11.078  -0.461   0.858   0.002   0.139
E Q  1DFJI                 24    0.554  98.909   0.946   0.502   0.002   0.495
E Q  1DFJI                 25    0.554  98.873   1.589   0.321   0.003   0.675
B Y  1DFJI                 26    0.071  15.151   0.929   0.181   0.016   0.803
E E  1DFJI                 27    0.423  73.881   1.129   0.018   0.088   0.893
E V  1DFJI                 28    0.385  59.205   1.596   0.004   0.197   0.799
B V  1DFJI                 29    0.042   6.486   0.076   0.001   0.900   0.099
E R  1DFJI                 30    0.295  67.532   1.081   0.001   0.900   0.099
B L  1DFJI                 31    0.043   7.910  -0.746   0.001   0.900   0.099
E D  1DFJI                 32    0.395  56.862  -0.212   0.023   0.655   0.322
E D  1DFJI                 33    0.596  85.884  -0.745   0.004   0.420   0.576
B C  1DFJI                 34    0.136  19.066  -0.359   0.005   0.336   0.660
E G  1DFJI                 35    0.480  37.745   0.090   0.005   0.262   0.733
B L  1DFJI                 36    0.050   9.063  -0.280   0.005   0.262   0.733
E T  1DFJI                 37    0.317  44.010   0.715   0.004   0.138   0.858
E E  1DFJI                 38    0.540  94.338  -1.531   0.018   0.019   0.964
E E  1DFJI                 39    0.629 109.904   0.838   0.782   0.003   0.216
B H  1DFJI                 40    0.125  22.756  -0.467   0.782   0.003   0.216
B C  1DFJI                 41    0.047   6.641   0.082   0.782   0.003   0.216
E K  1DFJI                 42    0.467  96.124   1.032   0.923   0.002   0.076
E D  1DFJI                 43    0.279  40.218   0.789   0.923   0.002   0.076
B I  1DFJI                 44    0.021   3.885   1.220   0.970   0.001   0.030
B G  1DFJI                 45    0.087   6.871  -0.873   0.970   0.001   0.030
E S  1DFJI                 46    0.499  58.495   1.088   0.970   0.001   0.030
B A  1DFJI                 47    0.085   9.378  -0.967   0.970   0.001   0.030
B L  1DFJI                 48    0.040   7.342  -1.621   0.970   0.001   0.030
E R  1DFJI                 49    0.519 118.805   0.865   0.858   0.002   0.139
E A  1DFJI                 50    0.669  73.691   0.924   0.694   0.003   0.303
B N  1DFJI                 51    0.122  17.919   0.216   0.406   0.004   0.590
E P  1DFJI                 52    0.602  85.381   0.237   0.053   0.005   0.942
E S  1DFJI                 53    0.500  58.635   1.485   0.018   0.019   0.964
B L  1DFJI                 54    0.070  12.854   0.516   0.004   0.085   0.910
E T  1DFJI                 55    0.396  54.995   1.519   0.004   0.197   0.799
E E  1DFJI                 56    0.431  75.296   1.509   0.005   0.336   0.660
B L  1DFJI                 57    0.039   7.123   0.793   0.001   0.900   0.099
E C  1DFJI                 58    0.310  43.580   0.838   0.001   0.959   0.040
B L  1DFJI                 59    0.057  10.492   0.060   0.001   0.900   0.099
E R  1DFJI                 60    0.417  95.447  -0.335   0.004   0.514   0.481
E T  1DFJI                 61    0.617  85.550  -1.355   0.005   0.336   0.660
B N  1DFJI                 62    0.197  28.885  -0.177   0.004   0.138   0.858
E E  1DFJI                 63    0.524  91.525  -0.144   0.004   0.138   0.858
B L  1DFJI                 64    0.068  12.487  -1.199   0.004   0.138   0.858
E G  1DFJI                 65    0.317  24.956  -0.011   0.018   0.047   0.935
E D  1DFJI                 66    0.513  73.880  -1.675   0.018   0.019   0.964
E A  1DFJI                 67    0.630  69.415   0.608   0.694   0.003   0.303
B G  1DFJI                 68    0.099   7.831  -0.227   0.858   0.002   0.139
B V  1DFJI                 69    0.035   5.380  -0.278   0.858   0.002   0.139
E H  1DFJI                 70    0.467  84.947   1.311   0.970   0.001   0.030
E L  1DFJI                 71    0.310  56.688   0.987   0.970   0.001   0.030
B V  1DFJI                 72    0.023   3.581   0.760   0.970   0.001   0.030
B L  1DFJI                 73    0.054   9.924  -0.316   0.970   0.001   0.030
E Q  1DFJI                 74    0.554  98.944   1.420   0.970   0.001   0.030
B G  1DFJI                 75    0.143  11.286  -0.438   0.970   0.001   0.030
B L  1DFJI                 76    0.056  10.309  -0.235   0.970   0.001   0.030
E Q  1DFJI                 77    0.502  89.604   0.161   0.923   0.002   0.076
E S  1DFJI                 78    0.614  72.008   0.031   0.782   0.003   0.216
E P  1DFJI                 79    0.366  51.907  -1.113   0.502   0.002   0.495
E T  1DFJI                 80    0.502  69.572  -1.207   0.176   0.004   0.820
B C  1DFJI                 81    0.305  42.794  -0.546   0.018   0.019   0.964
E K  1DFJI                 82    0.443  91.166   1.060   0.018   0.047   0.935
B I  1DFJI                 83    0.084  15.577   0.045   0.019   0.141   0.840
E Q  1DFJI                 84    0.330  58.956   0.709   0.021   0.279   0.699
E K  1DFJI                 85    0.356  73.147   1.291   0.004   0.514   0.481
B L  1DFJI                 86    0.038   6.994   0.104   0.001   0.900   0.099
E S  1DFJI                 87    0.247  28.972   0.783   0.001   0.959   0.040
B L  1DFJI                 88    0.039   7.232  -0.935   0.001   0.900   0.099
E Q  1DFJI                 89    0.319  56.938  -0.439   0.023   0.655   0.322
E N  1DFJI                 90    0.513  75.147  -0.761   0.022   0.359   0.619
B C  1DFJI                 91    0.101  14.223  -1.295   0.021   0.279   0.699
E S  1DFJI                 92    0.433  50.724   0.175   0.004   0.197   0.799
B L  1DFJI                 93    0.040   7.251  -0.874   0.004   0.197   0.799
E T  1DFJI                 94    0.305  42.345   0.797   0.004   0.085   0.910
E E  1DFJI                 95    0.584 102.095  -1.613   0.018   0.019   0.964
E A  1DFJI                 96    0.650  71.652   0.888   0.782   0.003   0.216
B G  1DFJI                 97    0.117   9.216  -0.356   0.858   0.002   0.139
B C  1DFJI                 98    0.052   7.273  -0.019   0.858   0.002   0.139
E G  1DFJI                 99    0.441  34.667   1.091   0.923   0.002   0.076
E V  1DFJI                100    0.319  49.076   1.077   0.970   0.001   0.030
B L  1DFJI                101    0.026   4.724   0.913   0.970   0.001   0.030
B P  1DFJI                102    0.083  11.778  -0.608   0.970   0.001   0.030
E S  1DFJI                103    0.542  63.522   1.213   0.970   0.001   0.030
B T  1DFJI                104    0.128  17.712  -0.756   0.970   0.001   0.030
B L  1DFJI                105    0.059  10.785  -0.753   0.923   0.002   0.076
E R  1DFJI                106    0.513 117.546   0.721   0.858   0.002   0.139
E S  1DFJI                107    0.657  77.047   0.694   0.694   0.003   0.303
B L  1DFJI                108    0.122  22.357   0.288   0.406   0.004   0.590
E P  1DFJI                109    0.567  80.401   0.426   0.053   0.005   0.942
E T  1DFJI                110    0.430  59.669   1.660   0.018   0.019   0.964
B L  1DFJI                111    0.048   8.844   0.652   0.004   0.085   0.910
E R  1DFJI                112    0.368  84.341   1.466   0.004   0.197   0.799
E E  1DFJI                113    0.421  73.636   1.481   0.005   0.336   0.660
B L  1DFJI                114    0.044   8.111   1.153   0.001   0.900   0.099
E H  1DFJI                115    0.308  56.080   0.679   0.001   0.959   0.040
B L  1DFJI                116    0.054   9.814  -0.244   0.001   0.900   0.099
E S  1DFJI                117    0.369  43.294  -0.334   0.004   0.420   0.576
E D  1DFJI                118    0.615  88.665  -0.725   0.005   0.262   0.733
B N  1DFJI                119    0.146  21.389  -0.268   0.004   0.138   0.858
E P  1DFJI                120    0.550  78.002   0.341   0.004   0.138   0.858
B L  1DFJI                121    0.083  15.289  -1.171   0.019   0.141   0.840
E G  1DFJI                122    0.366  28.804  -0.286   0.018   0.047   0.935
E D  1DFJI                123    0.562  80.941  -1.790   0.018   0.019   0.964
E A  1DFJI                124    0.615  67.817   0.720   0.694   0.003   0.303
B G  1DFJI                125    0.108   8.476  -0.469   0.782   0.003   0.216
B L  1DFJI                126    0.039   7.141  -0.300   0.858   0.002   0.139
E R  1DFJI                127    0.445 101.859   1.396   0.970   0.001   0.030
E L  1DFJI                128    0.274  50.188   0.879   0.970   0.001   0.030
B L  1DFJI                129    0.026   4.779   0.526   0.970   0.001   0.030
B C  1DFJI                130    0.053   7.427  -0.675   0.970   0.001   0.030
E E  1DFJI                131    0.550  95.998   1.103   0.970   0.001   0.030
B G  1DFJI                132    0.153  12.073  -1.140   0.970   0.001   0.030
B L  1DFJI                133    0.075  13.751  -1.594   0.923   0.002   0.076
E L  1DFJI                134    0.504  92.282  -0.141   0.923   0.002   0.076
E D  1DFJI                135    0.620  89.385  -0.470   0.782   0.003   0.216
E P  1DFJI                136    0.348  49.324  -0.885   0.502   0.002   0.495
E Q  1DFJI                137    0.474  84.621  -1.240   0.246   0.004   0.750
B C  1DFJI                138    0.277  38.877  -0.353   0.058   0.017   0.925
E H  1DFJI                139    0.444  80.764   0.824   0.052   0.084   0.864
B L  1DFJI                140    0.132  24.261  -0.117   0.056   0.142   0.802
E E  1DFJI                141    0.300  52.323   0.834   0.022   0.359   0.619
E K  1DFJI                142    0.383  78.824   1.419   0.004   0.514   0.481
B L  1DFJI                143    0.038   7.013   0.271   0.001   0.900   0.099
E Q  1DFJI                144    0.274  48.919   1.116   0.001   0.959   0.040
B L  1DFJI                145    0.056  10.290  -0.138   0.001   0.959   0.040
E E  1DFJI                146    0.373  65.128  -0.332   0.003   0.718   0.279
E Y  1DFJI                147    0.501 107.128  -0.731   0.004   0.616   0.381
B C  1DFJI                148    0.123  17.311  -0.429   0.004   0.420   0.576
E R  1DFJI                149    0.459 105.111  -0.020   0.005   0.336   0.660
B L  1DFJI                150    0.053   9.778  -0.224   0.005   0.336   0.660
E T  1DFJI                151    0.305  42.290   0.525   0.004   0.085   0.910
E A  1DFJI                152    0.604  66.561  -1.622   0.018   0.019   0.964
E A  1DFJI                153    0.659  72.578   0.597   0.600   0.003   0.397
B S  1DFJI                154    0.143  16.783  -0.607   0.600   0.003   0.397
B C  1DFJI                155    0.068   9.533  -0.111   0.502   0.002   0.495
E E  1DFJI                156    0.498  86.931   0.781   0.858   0.002   0.139
E P  1DFJI                157    0.292  41.477   0.802   0.923   0.002   0.076
B L  1DFJI                158    0.025   4.651   0.982   0.923   0.002   0.076
B A  1DFJI                159    0.077   8.430  -0.846   0.970   0.001   0.030
E S  1DFJI                160    0.526  61.659   1.106   0.970   0.001   0.030
B V  1DFJI                161    0.100  15.431  -0.963   0.970   0.001   0.030
B L  1DFJI                162    0.051   9.265  -0.698   0.970   0.001   0.030
E R  1DFJI                163    0.530 121.278   0.440   0.923   0.002   0.076
E A  1DFJI                164    0.665  73.239   0.676   0.782   0.003   0.216
B T  1DFJI                165    0.103  14.342  -0.010   0.406   0.004   0.590
E R  1DFJI                166    0.593 135.774   0.133   0.053   0.005   0.942
E A  1DFJI                167    0.439  48.356   1.442   0.005   0.015   0.979
B L  1DFJI                168    0.053   9.778   0.187   0.005   0.045   0.951
E K  1DFJI                169    0.370  76.191   1.178   0.004   0.085   0.910
E E  1DFJI                170    0.431  75.278   1.378   0.005   0.262   0.733
B L  1DFJI                171    0.037   6.775   0.590   0.001   0.900   0.099
E T  1DFJI                172    0.287  39.765   0.814   0.001   0.900   0.099
B V  1DFJI                173    0.059   9.068  -0.111   0.002   0.816   0.182
E S  1DFJI                174    0.375  43.915  -0.521   0.004   0.420   0.576
E N  1DFJI                175    0.579  84.722  -0.906   0.005   0.262   0.733
B N  1DFJI                176    0.200  29.295  -0.637   0.018   0.088   0.893
E D  1DFJI                177    0.544  78.434   0.225   0.004   0.085   0.910
B I  1DFJI                178    0.071  13.135  -1.895   0.019   0.141   0.840
E G  1DFJI                179    0.311  24.507  -0.144   0.018   0.088   0.893
E E  1DFJI                180    0.532  92.958  -1.422   0.058   0.017   0.925
E A  1DFJI                181    0.630  69.459   0.858   0.782   0.003   0.216
B G  1DFJI                182    0.095   7.469  -0.163   0.858   0.002   0.139
B A  1DFJI                183    0.033   3.637  -0.460   0.858   0.002   0.139
E R  1DFJI                184    0.476 108.935   1.420   0.970   0.001   0.030
E V  1DFJI                185    0.315  48.369   0.838   0.970   0.001   0.030
B L  1DFJI                186    0.028   5.182   0.316   0.970   0.001   0.030
B G  1DFJI                187    0.066   5.171  -1.053   0.970   0.001   0.030
E Q  1DFJI                188    0.569 101.713   1.419   0.970   0.001   0.030
B G  1DFJI                189    0.144  11.333  -0.865   0.970   0.001   0.030
B L  1DFJI                190    0.070  12.909  -1.056   0.970   0.001   0.030
E A  1DFJI                191    0.554  61.106  -0.048   0.923   0.002   0.076
E D  1DFJI                192    0.642  92.469  -0.037   0.782   0.003   0.216
E S  1DFJI                193    0.303  35.453  -1.303   0.502   0.002   0.495
E A  1DFJI                194    0.452  49.810  -1.113   0.176   0.004   0.820
B C  1DFJI                195    0.293  41.123  -0.826   0.058   0.017   0.925
E Q  1DFJI                196    0.432  77.137   0.746   0.053   0.043   0.903
B L  1DFJI                197    0.114  20.818  -0.263   0.056   0.142   0.802
E E  1DFJI                198    0.305  53.301   0.718   0.021   0.279   0.699
E T  1DFJI                199    0.378  52.387   1.516   0.022   0.552   0.426
B L  1DFJI                200    0.032   5.932   0.215   0.001   0.900   0.099
E R  1DFJI                201    0.281  64.395   1.030   0.011   0.918   0.071
B L  1DFJI                202    0.052   9.521  -0.183   0.011   0.918   0.071
E E  1DFJI                203    0.378  66.054  -0.654   0.022   0.552   0.426
E N  1DFJI                204    0.588  86.142  -0.582   0.020   0.205   0.775
B C  1DFJI                205    0.112  15.725  -1.086   0.019   0.141   0.840
E G  1DFJI                206    0.450  35.439  -0.090   0.004   0.085   0.910
B L  1DFJI                207    0.050   9.137  -0.747   0.004   0.138   0.858
E T  1DFJI                208    0.306  42.456   0.377   0.004   0.085   0.910
E P  1DFJI                209    0.551  78.130  -1.447   0.018   0.019   0.964
E A  1DFJI                210    0.613  67.497   0.589   0.782   0.003   0.216
B N  1DFJI                211    0.100  14.596  -0.454   0.782   0.003   0.216
B C  1DFJI                212    0.046   6.515  -0.208   0.858   0.002   0.139
E K  1DFJI                213    0.482  99.189   0.932   0.923   0.002   0.076
E D  1DFJI                214    0.341  49.153   0.892   0.970   0.001   0.030
B L  1DFJI                215    0.026   4.706   0.870   0.970   0.001   0.030
B C  1DFJI                216    0.110  15.500  -0.608   0.970   0.001   0.030
E G  1DFJI                217    0.546  42.939   1.185   0.970   0.001   0.030
B I  1DFJI                218    0.098  18.222  -0.916   0.970   0.001   0.030
B V  1DFJI                219    0.059   9.099  -0.822   0.970   0.001   0.030
E A  1DFJI                220    0.539  59.376   0.667   0.923   0.002   0.076
E S  1DFJI                221    0.664  77.821   0.819   0.782   0.003   0.216
B Q  1DFJI                222    0.125  22.361   0.144   0.502   0.002   0.495
E A  1DFJI                223    0.569  62.693   0.485   0.109   0.005   0.886
E S  1DFJI                224    0.439  51.486   1.663   0.018   0.019   0.964
B L  1DFJI                225    0.049   9.009   0.313   0.018   0.088   0.893
E R  1DFJI                226    0.380  87.089   1.421   0.004   0.138   0.858
E E  1DFJI                227    0.419  73.217   1.580   0.004   0.197   0.799
B L  1DFJI                228    0.040   7.379   0.417   0.001   0.900   0.099
E D  1DFJI                229    0.274  39.483   0.740   0.001   0.900   0.099
B L  1DFJI                230    0.056  10.327  -0.257   0.018   0.846   0.136
E G  1DFJI                231    0.354  27.836  -0.754   0.021   0.451   0.528
E S  1DFJI                232    0.554  64.917  -0.523   0.021   0.279   0.699
B N  1DFJI                233    0.135  19.749  -0.647   0.019   0.141   0.840
E G  1DFJI                234    0.517  40.696   0.057   0.019   0.141   0.840
B L  1DFJI                235    0.097  17.706  -1.013   0.019   0.141   0.840
E G  1DFJI                236    0.357  28.064  -0.144   0.018   0.088   0.893
E D  1DFJI                237    0.545  78.477  -2.045   0.058   0.017   0.925
E A  1DFJI                238    0.630  69.437   0.669   0.600   0.003   0.397
B G  1DFJI                239    0.104   8.177  -0.237   0.782   0.003   0.216
B I  1DFJI                240    0.032   5.846  -0.130   0.858   0.002   0.139
E A  1DFJI                241    0.453  49.899   1.139   0.923   0.002   0.076
E E  1DFJI                242    0.337  58.839   0.536   0.970   0.001   0.030
B L  1DFJI                243    0.032   5.804   0.063   0.970   0.001   0.030
B C  1DFJI                244    0.058   8.073  -0.828   0.970   0.001   0.030
E P  1DFJI                245    0.546  77.435   1.031   0.970   0.001   0.030
B G  1DFJI                246    0.164  12.891  -1.555   0.970   0.001   0.030
B L  1DFJI                247    0.074  13.641  -1.312   0.970   0.001   0.030
E L  1DFJI                248    0.508  93.015  -0.033   0.923   0.002   0.076
E S  1DFJI                249    0.672  78.794  -0.075   0.858   0.002   0.139
E P  1DFJI                250    0.442  62.748  -0.927   0.502   0.002   0.495
B A  1DFJI                251    0.291  32.079  -0.766   0.246   0.004   0.750
E S  1DFJI                252    0.387  45.368  -0.118   0.018   0.019   0.964
E R  1DFJI                253    0.412  94.440   0.721   0.018   0.047   0.935
B L  1DFJI                254    0.097  17.687   0.561   0.019   0.141   0.840
E K  1DFJI                255    0.286  58.830   0.883   0.005   0.262   0.733
E T  1DFJI                256    0.365  50.695   1.541   0.004   0.420   0.576
B L  1DFJI                257    0.028   5.163   0.598   0.001   0.900   0.099
E W  1DFJI                258    0.274  65.993   1.042   0.001   0.959   0.040
B L  1DFJI                259    0.037   6.756  -1.199   0.001   0.900   0.099
E W  1DFJI                260    0.332  79.774  -0.750   0.004   0.616   0.381
E E  1DFJI                261    0.555  97.011  -0.597   0.005   0.336   0.660
B C  1DFJI                262    0.090  12.664  -1.039   0.004   0.197   0.799
E D  1DFJI                263    0.438  63.159  -0.066   0.004   0.197   0.799
B I  1DFJI                264    0.044   8.122  -0.974   0.004   0.197   0.799
E T  1DFJI                265    0.322  44.661   0.765   0.004   0.085   0.910
E A  1DFJI                266    0.627  69.084  -1.496   0.018   0.019   0.964
E S  1DFJI                267    0.645  75.535   0.497   0.600   0.003   0.397
B G  1DFJI                268    0.122   9.570  -0.425   0.694   0.003   0.303
B C  1DFJI                269    0.050   6.978  -0.360   0.694   0.003   0.303
E R  1DFJI                270    0.477 109.302   0.807   0.858   0.002   0.139
E D  1DFJI                271    0.277  39.844   0.684   0.923   0.002   0.076
B L  1DFJI                272    0.022   4.083   1.211   0.923   0.002   0.076
B C  1DFJI                273    0.072  10.151  -0.620   0.970   0.001   0.030
E R  1DFJI                274    0.533 122.149   1.164   0.970   0.001   0.030
B V  1DFJI                275    0.107  16.415  -1.138   0.970   0.001   0.030
B L  1DFJI                276    0.054   9.924  -0.710   0.923   0.002   0.076
E Q  1DFJI                277    0.522  93.283   0.300   0.858   0.002   0.139
E A  1DFJI                278    0.669  73.724   0.658   0.600   0.003   0.397
B K  1DFJI                279    0.100  20.591   0.252   0.406   0.004   0.590
E E  1DFJI                280    0.563  98.356   0.528   0.053   0.005   0.942
E T  1DFJI                281    0.407  56.520   1.604   0.018   0.019   0.964
B L  1DFJI                282    0.050   9.155   0.603   0.018   0.047   0.935
E K  1DFJI                283    0.338  69.465   1.157   0.018   0.088   0.893
E E  1DFJI                284    0.422  73.741   1.609   0.004   0.197   0.799
B L  1DFJI                285    0.038   6.976   0.371   0.002   0.816   0.182
B S  1DFJI                286    0.269  31.480   0.672   0.011   0.918   0.071
B L  1DFJI                287    0.049   8.917  -0.501   0.018   0.846   0.136
E A  1DFJI                288    0.319  35.198  -0.728   0.022   0.359   0.619
E G  1DFJI                289    0.565  44.442  -0.371   0.020   0.205   0.775
B N  1DFJI                290    0.104  15.240  -0.624   0.019   0.141   0.840
E K  1DFJI                291    0.478  98.242   0.166   0.019   0.141   0.840
B L  1DFJI                292    0.058  10.638  -0.753   0.020   0.205   0.775
E G  1DFJI                293    0.374  29.426  -0.080   0.018   0.088   0.893
E D  1DFJI                294    0.498  71.719  -2.152   0.018   0.019   0.964
E E  1DFJI                295    0.581 101.466   0.448   0.406   0.004   0.590
B G  1DFJI                296    0.111   8.720  -0.833   0.694   0.003   0.303
B A  1DFJI                297    0.041   4.474  -0.540   0.782   0.003   0.216
E R  1DFJI                298    0.407  93.249   0.881   0.923   0.002   0.076
E L  1DFJI                299    0.271  49.547   0.482   0.970   0.001   0.030
B L  1DFJI                300    0.028   5.072   0.566   0.970   0.001   0.030
B C  1DFJI                301    0.052   7.315  -0.560   0.970   0.001   0.030
E E  1DFJI                302    0.497  86.808   0.781   0.970   0.001   0.030
B S  1DFJI                303    0.137  16.068  -1.155   0.970   0.001   0.030
B L  1DFJI                304    0.077  14.154  -1.813   0.970   0.001   0.030
E L  1DFJI                305    0.501  91.678  -0.703   0.923   0.002   0.076
E Q  1DFJI                306    0.628 112.143  -0.714   0.782   0.003   0.216
E P  1DFJI                307    0.423  59.953  -1.282   0.406   0.004   0.590
E G  1DFJI                308    0.378  29.741  -0.959   0.246   0.004   0.750
B C  1DFJI                309    0.324  45.433  -0.520   0.018   0.019   0.964
E Q  1DFJI                310    0.414  73.923   0.856   0.018   0.047   0.935
B L  1DFJI                311    0.112  20.434   0.255   0.019   0.141   0.840
E E  1DFJI                312    0.246  42.994   0.645   0.021   0.279   0.699
E S  1DFJI                313    0.353  41.407   1.284   0.004   0.420   0.576
B L  1DFJI                314    0.030   5.475   0.517   0.001   0.900   0.099
E W  1DFJI                315    0.261  62.795   0.500   0.001   0.959   0.040
B V  1DFJI                316    0.036   5.456  -0.982   0.011   0.918   0.071
E K  1DFJI                317    0.309  63.520  -0.627   0.022   0.552   0.426
E S  1DFJI                318    0.506  59.268  -0.978   0.005   0.262   0.733
B C  1DFJI                319    0.083  11.667  -0.934   0.004   0.197   0.799
E S  1DFJI                320    0.437  51.181  -0.054   0.004   0.138   0.858
B L  1DFJI                321    0.043   7.928  -0.983   0.004   0.138   0.858
E T  1DFJI                322    0.308  42.706   0.699   0.018   0.088   0.893
E A  1DFJI                323    0.537  59.144  -1.463   0.018   0.019   0.964
E A  1DFJI                324    0.604  66.528   0.320   0.694   0.003   0.303
B C  1DFJI                325    0.085  11.962  -0.479   0.694   0.003   0.303
B C  1DFJI                326    0.045   6.318  -0.118   0.782   0.003   0.216
E Q  1DFJI                327    0.477  85.121   0.760   0.923   0.002   0.076
E H  1DFJI                328    0.298  54.279   0.739   0.923   0.002   0.076
B V  1DFJI                329    0.027   4.119   0.976   0.923   0.002   0.076
B S  1DFJI                330    0.073   8.556  -0.662   0.970   0.001   0.030
E L  1DFJI                331    0.520  95.267   1.162   0.970   0.001   0.030
B M  1DFJI                332    0.088  17.689  -1.608   0.970   0.001   0.030
B L  1DFJI                333    0.052   9.503  -0.986   0.970   0.001   0.030
E T  1DFJI                334    0.504  69.946   0.357   0.923   0.002   0.076
E Q  1DFJI                335    0.681 121.609   0.861   0.782   0.003   0.216
B N  1DFJI                336    0.094  13.747   0.274   0.502   0.002   0.495
E K  1DFJI                337    0.591 121.589   0.656   0.053   0.005   0.942
E H  1DFJI                338    0.404  73.524   1.716   0.018   0.019   0.964
B L  1DFJI                339    0.043   7.855   0.404   0.005   0.045   0.951
E L  1DFJI                340    0.336  61.558   1.388   0.004   0.085   0.910
E E  1DFJI                341    0.440  76.868   1.650   0.005   0.262   0.733
B L  1DFJI                342    0.044   8.075   1.000   0.001   0.900   0.099
E Q  1DFJI                343    0.314  56.027   0.376   0.001   0.959   0.040
B L  1DFJI                344    0.053   9.631   0.018   0.001   0.900   0.099
E S  1DFJI                345    0.378  44.348  -0.552   0.005   0.336   0.660
E S  1DFJI                346    0.591  69.253  -0.258   0.004   0.197   0.799
B N  1DFJI                347    0.140  20.569  -0.553   0.004   0.085   0.910
E K  1DFJI                348    0.514 105.833   0.748   0.004   0.138   0.858
B L  1DFJI                349    0.056  10.272  -0.376   0.021   0.279   0.699
E G  1DFJI                350    0.345  27.120   0.406   0.019   0.141   0.840
E D  1DFJI                351    0.572  82.396  -2.123   0.018   0.019   0.964
E S  1DFJI                352    0.604  70.812   0.374   0.406   0.004   0.590
B G  1DFJI                353    0.101   7.980  -0.332   0.694   0.003   0.303
B I  1DFJI                354    0.036   6.568  -0.125   0.782   0.003   0.216
E Q  1DFJI                355    0.426  76.012   1.256   0.923   0.002   0.076
E E  1DFJI                356    0.295  51.536   1.159   0.970   0.001   0.030
B L  1DFJI                357    0.022   3.937   0.937   0.970   0.001   0.030
B C  1DFJI                358    0.080  11.162  -0.915   0.970   0.001   0.030
E Q  1DFJI                359    0.553  98.748   1.216   0.970   0.001   0.030
B A  1DFJI                360    0.102  11.295  -0.999   0.970   0.001   0.030
B L  1DFJI                361    0.054   9.979  -0.087   0.923   0.002   0.076
E S  1DFJI                362    0.559  65.468   0.536   0.923   0.002   0.076
E Q  1DFJI                363    0.643 114.876   0.402   0.782   0.003   0.216
E P  1DFJI                364    0.342  48.544  -0.720   0.502   0.002   0.495
E G  1DFJI                365    0.513  40.342  -1.140   0.246   0.004   0.750
B T  1DFJI                366    0.300  41.624  -0.195   0.018   0.019   0.964
E T  1DFJI                367    0.419  58.143   1.028   0.053   0.043   0.903
B L  1DFJI                368    0.098  17.889   0.497   0.019   0.141   0.840
E R  1DFJI                369    0.278  63.685   0.975   0.005   0.336   0.660
E V  1DFJI                370    0.354  54.441   1.279   0.004   0.514   0.481
B L  1DFJI                371    0.033   6.116   0.614   0.001   0.959   0.040
B C  1DFJI                372    0.243  34.061   0.430   0.001   0.959   0.040
B L  1DFJI                373    0.049   9.045  -0.088   0.001   0.900   0.099
E G  1DFJI                374    0.347  27.340  -0.585   0.004   0.616   0.381
E D  1DFJI                375    0.550  79.255  -0.545   0.005   0.262   0.733
B C  1DFJI                376    0.097  13.577  -1.082   0.004   0.197   0.799
E E  1DFJI                377    0.449  78.458   0.386   0.004   0.138   0.858
B V  1DFJI                378    0.038   5.917  -1.304   0.004   0.197   0.799
E T  1DFJI                379    0.318  44.107   0.580   0.004   0.085   0.910
E N  1DFJI                380    0.547  80.052  -1.326   0.018   0.019   0.964
E S  1DFJI                381    0.632  74.094   0.992   0.694   0.003   0.303
B G  1DFJI                382    0.111   8.728  -0.709   0.782   0.003   0.216
B C  1DFJI                383    0.043   6.051   0.156   0.782   0.003   0.216
E S  1DFJI                384    0.470  55.131   1.096   0.923   0.002   0.076
E S  1DFJI                385    0.310  36.285   0.963   0.923   0.002   0.076
B L  1DFJI                386    0.026   4.779   0.981   0.970   0.001   0.030
B A  1DFJI                387    0.103  11.362  -0.592   0.970   0.001   0.030
E S  1DFJI                388    0.554  64.905   1.356   0.970   0.001   0.030
B L  1DFJI                389    0.089  16.333  -1.634   0.970   0.001   0.030
B L  1DFJI                390    0.068  12.378  -0.825   0.970   0.001   0.030
E L  1DFJI                391    0.547 100.101   0.618   0.923   0.002   0.076
E A  1DFJI                392    0.659  72.578   0.916   0.782   0.003   0.216
B N  1DFJI                393    0.117  17.173   0.142   0.502   0.002   0.495
E R  1DFJI                394    0.585 133.965   0.665   0.053   0.005   0.942
E S  1DFJI                395    0.451  52.916   1.839   0.018   0.019   0.964
B L  1DFJI                396    0.048   8.880   0.603   0.004   0.085   0.910
E R  1DFJI                397    0.349  79.990   1.182   0.004   0.138   0.858
E E  1DFJI                398    0.438  76.588   1.486   0.004   0.197   0.799
B L  1DFJI                399    0.037   6.756   0.669   0.001   0.900   0.099
E D  1DFJI                400    0.291  41.875   0.712   0.001   0.959   0.040
B L  1DFJI                401    0.042   7.617  -0.555   0.001   0.900   0.099
E S  1DFJI                402    0.355  41.618  -0.637   0.004   0.420   0.576
E N  1DFJI                403    0.547  80.110  -1.088   0.004   0.197   0.799
B N  1DFJI                404    0.121  17.729  -1.235   0.004   0.085   0.910
E C  1DFJI                405    0.499  70.060   0.339   0.004   0.085   0.910
B V  1DFJI                406    0.050   7.716  -0.515   0.004   0.138   0.858
E G  1DFJI                407    0.325  25.585   0.220   0.005   0.045   0.951
E D  1DFJI                408    0.566  81.532  -1.637   0.016   0.005   0.979
E P  1DFJI                409    0.605  85.892   0.492   0.782   0.003   0.216
B G  1DFJI                410    0.073   5.761  -0.624   0.858   0.002   0.139
B V  1DFJI                411    0.043   6.548  -0.248   0.923   0.002   0.076
E L  1DFJI                412    0.441  80.729   1.236   0.970   0.001   0.030
E Q  1DFJI                413    0.290  51.740   0.893   0.970   0.001   0.030
B L  1DFJI                414    0.024   4.358   0.826   0.970   0.001   0.030
B L  1DFJI                415    0.087  16.021  -0.866   0.970   0.001   0.030
E G  1DFJI                416    0.529  41.601   1.269   0.970   0.001   0.030
B S  1DFJI                417    0.116  13.630  -1.471   0.970   0.001   0.030
B L  1DFJI                418    0.066  12.121  -1.061   0.970   0.001   0.030
E E  1DFJI                419    0.562  98.112   0.334   0.923   0.002   0.076
E Q  1DFJI                420    0.652 116.483   0.462   0.858   0.002   0.139
E P  1DFJI                421    0.384  54.475  -0.618   0.600   0.003   0.397
E G  1DFJI                422    0.459  36.123  -0.733   0.246   0.004   0.750
E C  1DFJI                423    0.416  58.336  -0.156   0.058   0.017   0.925
E A  1DFJI                424    0.387  42.647   1.228   0.053   0.043   0.903
B L  1DFJI                425    0.070  12.799   0.197   0.018   0.088   0.893
E E  1DFJI                426    0.376  65.652   0.987   0.020   0.205   0.775
E Q  1DFJI                427    0.408  72.887   1.710   0.005   0.336   0.660
B L  1DFJI                428    0.031   5.621   0.525   0.001   0.900   0.099
E V  1DFJI                429    0.314  48.231   0.860   0.001   0.900   0.099
B L  1DFJI                430    0.055  10.162  -0.460   0.001   0.900   0.099
E Y  1DFJI                431    0.409  87.425  -0.295   0.004   0.616   0.381
E D  1DFJI                432    0.557  80.321  -1.080   0.004   0.420   0.576
B T  1DFJI                433    0.158  21.915  -0.570   0.005   0.262   0.733
E Y  1DFJI                434    0.481 102.683   0.351   0.005   0.262   0.733
B W  1DFJI                435    0.061  14.670  -0.258   0.005   0.262   0.733
E T  1DFJI                436    0.328  45.466   0.207   0.018   0.088   0.893
E E  1DFJI                437    0.642 112.105  -0.983   0.058   0.017   0.925
E E  1DFJI                438    0.602 105.100   1.218   0.923   0.002   0.076
B V  1DFJI                439    0.060   9.299   0.006   0.923   0.002   0.076
B E  1DFJI                440    0.091  15.863   0.560   0.923   0.002   0.076
E D  1DFJI                441    0.550  79.197   1.341   0.970   0.001   0.030
B R  1DFJI                442    0.224  51.342   1.390   0.970   0.001   0.030
B L  1DFJI                443    0.018   3.314   1.242   0.970   0.001   0.030
E Q  1DFJI                444    0.269  48.026   0.919   0.970   0.001   0.030
E A  1DFJI                445    0.519  57.194   1.615   0.970   0.001   0.030
B L  1DFJI                446    0.063  11.609  -0.833   0.970   0.001   0.030
B E  1DFJI                447    0.165  28.843  -0.195   0.970   0.001   0.030
E G  1DFJI                448    0.634  49.888   0.863   0.923   0.002   0.076
E S  1DFJI                449    0.623  73.016   1.110   0.858   0.002   0.139
B K  1DFJI                450    0.151  31.122   0.762   0.694   0.003   0.303
E P  1DFJI                451    0.597  84.643   0.582   0.176   0.004   0.820
E G  1DFJI                452    0.524  41.215   1.737   0.053   0.005   0.942
B L  1DFJI                453    0.064  11.755   0.180   0.018   0.019   0.964
E R  1DFJI                454    0.525 120.179   1.698   0.018   0.047   0.935
E V  1DFJI                455    0.493  75.759   1.181   0.019   0.141   0.840
B I  1DFJI                456    0.180  33.337   0.225   0.066   0.296   0.638
E S  1DFJI                457    0.783  91.721  -1.517   0.053   0.043   0.903

Explain the output.  Go back.
```

---
